# Levels of Circulating PD-L1 Are Decreased in Patients with Resectable Cholangiocarcinoma

**DOI:** 10.3390/ijms22126569

**Published:** 2021-06-18

**Authors:** Christoph Roderburg, Sven H. Loosen, Jan Bednarsch, Patrick H. Alizai, Anjali A. Roeth, Sophia M. Schmitz, Mihael Vucur, Mark Luedde, Pia Paffenholz, Frank Tacke, Christian Trautwein, Tom F. Ulmer, Ulf Peter Neumann, Tom Luedde

**Affiliations:** 1 Department of Hepatology & Gastroenterology, Campus Virchow Klinikum and Campus Charité Mitte, Charité University Medicine Berlin, 13353 Berlin, Germany; frank.tacke@charite.de; 2 Clinic for Gastroenterology, Hepatology and Infectious Diseases, University Hospital Düsseldorf, Medical Faculty of Heinrich Heine University Düsseldorf, 40225 Düsseldorf, Germany; sven.loosen@med.uni-duesseldorf.de (S.H.L.); mihael.vucur@med.uni-duesseldorf.de (M.V.); tom.luedde@med.uni-duesseldorf.de (T.L.); 3 Department of Visceral and Transplantation Surgery, University Hospital RWTH Aachen, Pauwelsstrasse 30, 52074 Aachen, Germany; jbednarsch@ukaachen.de (J.B.); palizai@ukaachen.de (P.H.A.); aroeth@ukaachen.de (A.A.R.); sschmitz@ukaachen.de (S.M.S.); fulmer@ukaachen.de (T.F.U.); upneumann@ukaachen.de (U.P.N.); 4 KGP Bremerhaven, 27568 Bremerhaven, Germany; mark.luedde@web.de; 5 Department of Urology, University Hospital Cologne, Kerpener Straße 62, 50937 Cologne, Germany; pia.paffenholz@uk-koeln.de; 6 Department of Medicine III, University Hospital RWTH Aachen, Pauwelsstrasse 30, 52074 Aachen, Germany; ctrautwein@ukaachen.de

**Keywords:** biliary tract cancer, cholangiocarcinoma, PD-L1, PD-1, biomarker

## Abstract

Tumor resection represents the only curative treatment option for patients with biliary tract cancers (BTCs), including intrahepatic cholangiocarcinoma (CCA), perihilar and extrahepatic CCA and gallbladder cancer. However, many patients develop early tumor recurrence and are unlikely to benefit from surgery. Therefore, markers to identify ideal surgical candidates are urgently needed. Circulating programmed cell death 1 ligand 1 (PD-L1) has recently been associated with different malignancies, including pancreatic cancer which closely resembles BTC in terms of patients’ prognosis and tumor biology. Here, we aim at evaluating a potential role of circulating PD-L1 as a novel biomarker for resectable BTC. Methods: Serum levels of PD-L1 were analyzed by ELISA in 73 BTC patients and 42 healthy controls. Results: Circulating levels of preoperative PD-L1 were significantly lower in patients with BTC compared to controls. Patients with low PD-L1 levels displayed a strong trend towards an impaired prognosis, and circulating PD-L1 was negatively correlated with experimental markers of promalignant tumor characteristics such as CCL1, CCL21, CCL25 and CCL26. For 37 out of 73 patients, postoperative PD-L1 levels were available. Interestingly, after tumor resection, circulating PD-L1 raised to almost normal levels. Notably, patients with further decreasing PD-L1 concentrations after surgery showed a trend towards an impaired postoperative outcome. Conclusion: Circulating PD-L1 levels were decreased in patients with resectable BTC. Lack of normalization of PD-L1 levels after surgery might identify patients at high risk for tumor recurrence or adverse outcome.

## 1. Introduction

Biliary tract cancers, including intrahepatic cholangiocarcinoma, perihilar and extrahepatic CCA and gallbladder cancer, represent rare tumor entities accounting for up to 5% of all gastrointestinal malignancies [1]. Despite intensive research efforts, the prognosis of patients with BTC is still poor, and only complete tumor resection is able to provide long-term survival [2]. However, postoperative outcome following BTC resection is very heterogeneous, and many successfully resected patients (R0 resection) have rapid tumor recurrence [3]. Thus, a majority of patients undergoing curatively intended surgery are not likely to benefit from extensive liver surgery which is needed for complete tumor extirpation [3]. Unfortunately, at present, only very few single biomarkers or “scoring systems” are available for the identification of those patients that will particularly benefit from surgery and those that should rather be amended to other treatment modalities [4,5,6]. In this context, novel biomarkers that better reflect the individual tumor biology or the host’s antitumoral immune response and allow an estimation of the patients’ outcome after surgery are urgently needed.

PD-L1, representing a member of the B7 superfamily, is the ligand of the receptor programmed death-1 (PD-1), which is mainly expressed on T cells [7,8]. The binding of PD-L1 to PD-1 leads to the activation of PD-1, which in turn suppresses T-cell proliferation and reduces cytokine secretion, thereby reducing immune activation and allowing tumor cells to escape from immune recognition [7,8]. In many tumor entities, elevated expression levels of PD-L1 have been associated with an aggressive tumor biology and an unfavorable outcome [9]. Soluble PD-1/PD-L1 was suggested as a diagnostic, therapeutic and prognostic biomarker in different cancers (summarized in [10]), indicating, e.g., an impaired outcome and treatment response in patients with multiple myeloma [11]. Moreover, in a series of papers, different authors recently suggested a role of sPD-L1 as a biomarker in very different cancer entities and have suggested a diagnostic, prognostic and predictive role of this molecular marker in cancers originating from the urothelium, lung and brain [12,13,14]. In addition, sPD-L1 was found to be a prognostic marker in gastrointestinal stromal cancers and gastric cancers [15,16]. Therefore, evaluation of PD-L1 expression and/or PD-L1 serum levels as a surrogate for PD-L1 expression has been proposed as a valuable tool for the estimation of patients’ prognosis in manifold cancers. Just recently, the group of Park and colleges demonstrated that in pancreatic cancer, a tumor entity that closely resembles BTC in terms of prognosis and tumor biology, PD-L1 levels at diagnosis exhibit a prognostic value and that the course of PD-L1 concentrations correlates with clinical outcome [17]. In this study, we therefore evaluated a potential role of circulating PD-L1 as a diagnostic and/or prognostic biomarker in 73 BTC patients who underwent curative intended tumor-resection at our university hospital between 2011 and 2015.

## 2. Results

### 2.1. Concentrations of Circulating Preoperative PD-L1 Are Decreased in Patients with BTC at Diagnosis

**Figure 1:**
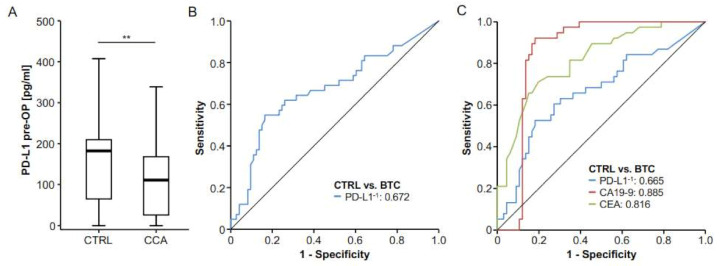
Serum levels of circulating PD-L1 are decreased in patients with BTC. (**A**) Serum levels of circulating PD-L1 are significantly lower in patients with BTC compared to healthy controls. (**B**) ROC curve analysis reveals an AUC value of 0.672 for circulating PD-L1 when measured for the differentiation between BTC and healthy controls. (**C**) Routinely measured BTC tumor markers showed higher AUC values for this setting (CEA: 0.816, CA19-9: 0.885); ** *p* < 0.01.

Based on previous results from different gastrointestinal malignancies [17,18,19,20], we hypothesized that serum levels of PD-L1 before surgery might also be altered in patients with resectable BTC. We therefore compared levels of circulating PD-L1 between patients with BTC (*n* = 73) and healthy controls (*n* = 42), revealing that BTC patients displayed significantly lower serum levels of PD-L1 compared to healthy controls (Figure 1A, Supplementary Table S1, 27/73 BTC patients and 10/42 of controls had an sPD-L1 level below the detection limit). We next tested the value of preoperative PD-L1 for the diagnosis of BTC. In this analysis, the diagnostic potential of circulating PD-L1 was rather limited, showing an AUC value of 0.672 (Figure 1B). At an ideal diagnostic cut-off value of 176.5 pg/mL, PD-L1 had a diagnostic sensitivity of 54.8% and a specificity of 83.6%. The diagnostic potential of circulating PD-L1 was inferior to established tumor markers such as CEA (AUC: 0.816) and CA19-9 (AUC: 0.885) when used as a single marker for the differentiation between BTC patients and healthy controls (Figure 1C).

**Figure 2:**
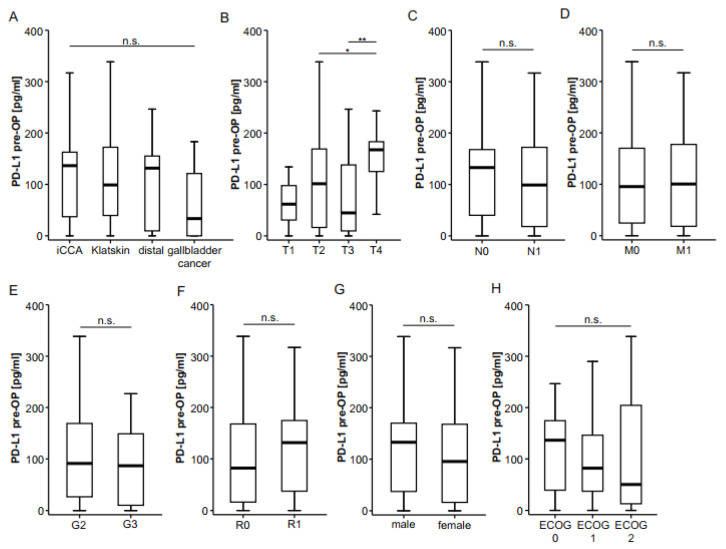
Preoperative serum PD-L1 does not correlate with clinicopathological characteristics. (**A**) Initial serum PD-L1 levels do not differ between different BTC localizations. (**B**) Concentrations of circulating PD-L1 are significantly higher in T4 tumors but unaltered between N0/N1 (**C**) and M0/M1 (**D**) stages. Concentrations of circulating PD-L1 are unaltered between moderately and poorly differentiated tumors (**E**), complete (R0) and incomplete tumor resection (R1, F), male and female patients (**G**) or patients with different ECOG performance status (**H**). n.s. not significant; * *p* < 0.05; ** *p* < 0.01.

We next examined serum concentrations of preoperative PD-L1 in patients with different tumor and patient characteristics in order to understand whether PD-L1 levels reflect tumor or disease stage in individual patients. Notably, PD-L1 concentrations did not vary between patients with different tumor localizations (Figure 2A) but were significantly higher in T4 patients (Figure 2B). PD-L1 serum levels did not differ in BTC patients with or without lymph node involvement (Figure 2C) or in nonmetastasized or metastasized patients who were still resectable (Figure 2D). Moreover, tumor grading (Figure 2E) or the resection status (Figure 2F) did not affect levels of circulating PD-L1 in our cohort of patients. Finally, preoperative PD-L1 concentrations were independent of sex (Figure 2G) or the patients’ ECOG performance status (Figure 2H).

In order to identify potential mechanisms influencing concentrations of circulating preoperative PD-L1 in patients with cholangiocarcinoma, we performed correlation analysis between PD-L1 and different markers routinely accessed in patients with BTC. However, preoperative PD-L1 did not correlate with levels of creatinine (r_s_ = −0.092, *p* = 0.440), bilirubin (r_s_ = 0.085, *p* = 0.480), aspartate aminotransferase (r_s_ = 0.068, *p* = 0.568), alanine aminotransferase (r_s_ = 0.086, *p* = 0.550), C-reactive protein (r_s_ = 0.126, *p* = 0.300), leukocyte count (r_s_ = −0.073, *p* = 0.543) or other standard laboratory parameters (Table 1).

### 2.2. Association of Preoperative PD-L1 Serum Concentrations and Patients’ Survival

**Figure 3:**
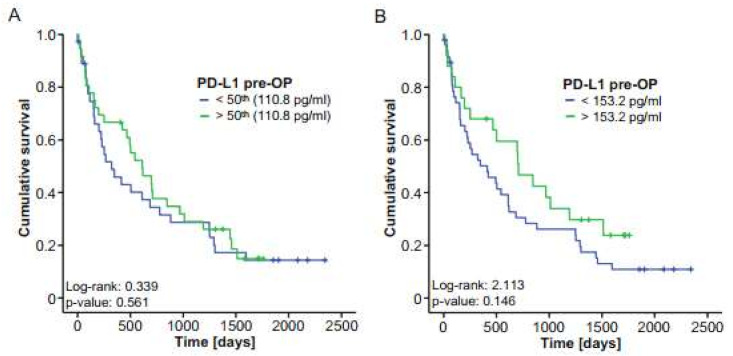
Initial serum PD-L1 does not reflect the outcome of BTC patients after tumor resection. (**A**) Kaplan–Meier curve analysis using the 50th percentile as a cut-off reveals no survival benefit for patients with high or low PD-L1 levels. (**B**) BTC patients with PD-L1 serum levels below the ideal prognostic cut-off value of 153.2 pg/mL show a nonsignificant trend towards an impaired survival. ** *p* < 0.01.

Concentrations of circulating PD-L1 were recently recognized as prognostic markers in manifold malignant diseases [10]. We therefore hypothesized that preoperative PD-L1 levels might also be indicative of the outcome of patients with BTC and performed Kaplan–Meier curve analysis using different cut-offs. However, survival in patients with PD-L1 levels above or below the 50th percentile was similar (Figure 3A). When using a statistically “optimal” cut-off value to distinguish between survivors and nonsurvivors, a trend towards a more favorable survival for patients with preoperative PD-L1 levels higher than 153.2 pg/mL became apparent; however, statistical significance was not reached (Figure 3B). In agreement, Cox regression analysis showed no significant survival benefit for patients with initial PD-L1 serum levels below or above 153.2 pg/mL (HR: 0.664 (0.381–1.158), *p*-value: 0.149). To further dissect potential underlying causes for this trend towards an impaired survival in patients with low initial PD-L1 levels, we measured serum levels of different cytokines (CCL1, CCL21, CCL25 and CCL26) that have been associated with promalignant characteristics [21,22,23,24] and correlated them with levels of circulating preoperative PD-L1 in our cohort of patients. Interestingly, we found highly significant negative correlations between initial PD-L1 and all of these chemokines, further corroborating the notion that circulating preoperative PD-L1 might have a prognostic function for resectable BTC (Supplementary Figure S2A–D).

### 2.3. Postoperative PD-L1 Concentrations Are Elevated in Patients with BTC After Tumor Resection

**Figure 4:**
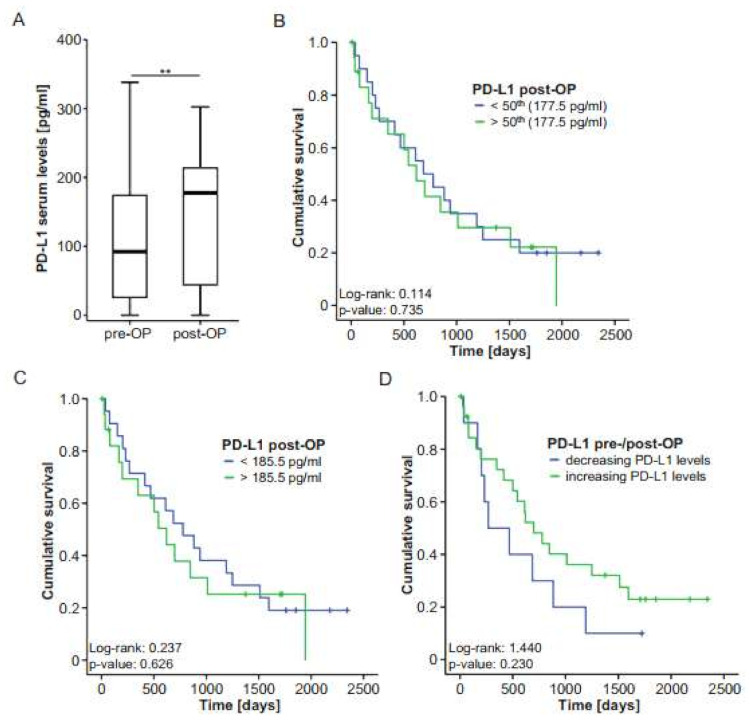
Postoperative PD-L1 levels do not predict outcome after BTC resection. (**A**) Postoperative PD-L1 serum levels are significantly higher compared to preoperative serum levels. (**B**,**C**) Kaplan–Meier curve analyses using the 50th percentile or the ideal prognostic cut-off value (185.5 pg/mL) reveal no survival benefit for patients with high or low PD-L1 levels. (**D**) BTC patients with decreasing PD-L1 serum levels after tumor resection show a nonsignificant trend towards an impaired survival. ** *p* < 0.01.

For *n* = 37 patients, PD-L1 concentrations at day 6 or 7 after tumor resection were available. We compared postoperative PD-L1 levels with the respective preoperative concentrations. In this analysis, we observed a significant increase after tumor resection to almost those levels measured in healthy controls (Figure 4A). Similar to preoperative levels, postoperative concentrations of PD-L1 were independent of patients’ or tumor characteristics such as different TNM stages (Supplementary Figure S1A–C), tumor grading (Supplementary Figure S1D), sex (Supplementary Figure S1E) and ECOG (Supplementary Figure S1F). Interestingly, patients with microscopically incomplete tumor resection (R1) displayed slightly elevated PD-L1 concentrations compared to patients with complete tumor resection (R0; Supplementary Figure S1G).

To analyze whether postoperative PD-L1 concentrations are indicative of the patients’ postoperative outcome, we compared the overall survival of patients with high or low postoperative PD-L1 levels (above or below the 50th percentile and above or below an ideal cut-off of 185.5 pg/mL) in Kaplan–Meier curve analysis. However, similar to our observations using initial PD-L1 levels, the survival of patients with high postoperative PD-L1 levels was similar to that of patients with low postoperative concentrations (Figure 4B,C). In agreement, Cox regression analysis showed no survival benefit for patients with postoperative PD-L1 serum levels below or above 185.5 pg/mL (HR: 1.197 (0.580–2.468), *p*-value: 0.627). Finally, we investigated whether the individual course of pre- and postoperative PD-L1 levels was linked with the patients’ survival. Notably, in this analysis, patients with increasing PD-L1 concentrations after tumor resection demonstrated a clear trend towards an improved survival compared to patients who displayed further decreasing PD-L1 levels after surgery (Figure 4D). This finding is in line with our previous observation that serum levels of PD-L1 are decreased in patients with BTC and that patients with lower PD-L1 concentrations demonstrate a trend towards an impaired survival after surgery.

## 3. Discussion

We demonstrate that serum levels of PD-L1 are significantly lower in patients with BTC when compared to healthy controls. While these data suggest a potential role of PD-L1 as a diagnostic marker in BTC, we failed to detect a significant prognostic function of circulating PD-L1 as PD-L1 serum concentrations reflected neither patients’ clinicopathological characteristics nor the individual postoperative outcome. Interestingly, tumor resection led to a restoration of PD-L1 levels to concentrations observed in healthy controls, suggesting that the low levels of PD-L1 at the time point of diagnosis directly reflect the presence of BTC in our cohort of patients. Notably, at least in our cohort of patients, the diagnostic value of sPD-L1 was smaller than that of classic biomarkers analyzed in the context of CCA such as CEA or CA-19-9, highlighting that a potential use of sPD-L1 as a diagnostic biomarker in clinical routine is rather unlikely. Moreover, the combinational use of sPD-L1 and CA19-9 and/or CEA did not significantly improve the diagnostic power as the AUC of the combination was similar or even lower when compared to CA19-9 or CEA alone.

In many cases, surgical tumor resection in patients with BTC is performed upon radiological suspicion in the absence of pathologic confirmation of diagnosis. In contrast, such a confirmation is required in patients with nonresectable tumor disease before a systemic therapy can be initiated [25]. However, pathological confirmation in the context of BTC might be challenging, particularly in patients affected by primary sclerosing cholangitis and biliary strictures. While cutting-edge endoscopic methods such as cholangioscopy have facilitated sample extraction, biopsy samples are often inadequate for molecular profiling, and in addition, tissue sampling has reported high specificity but low sensitivity in the diagnosis of malignant biliary strictures [25]. Finally, the highly desmoplastic nature of BTC limits the accuracy of cytological and pathological approaches [25]. Therefore, circulating biomarkers and especially liquid biopsy have gained growing attention over the years, given the promising applications in cancer patients. As an example, it was recently demonstrated that RNA profiles in serum and urine extracellular vesicles might mirror intratumoral gene expression and therefore be suitable for tumor diagnosis or guiding treatment decisions [26]. On the basis of these premises, our data might shed light on how diagnostic strategies in BTC might evolve in the next years.

BTC represents a lethal malignancy worldwide [1]. Despite intensive research efforts, patients with advanced disease stages face a limited prognosis, and treatment of these patients still relies on classical cytotoxic chemotherapy providing only marginal improvement of survival. In the era of immunotherapy, large randomized clinical trials have reported promising outcomes for anti-PD-1/PD-L1 directed therapies in patients with various cancers, such as lung cancer, melanoma and renal cell carcinoma [27,28]. However, in BTC, clinical trials investigating anti-PD-L1 directed therapies have shown disappointing results, even in PD-L1-positive patients [29]. Due to the very small numbers of BTC patients analyzed in immunotherapeutic studies so far, the role of circulating PD-L1 as a biomarker for BTC has not been evaluated in detail. Nevertheless, different authors have examined the prognostic and predictive function of PD-L1 in pancreatic cancer, which closely resembles BTC in terms of prognosis and biology. Tessier-Clouthier and colleagues demonstrated that high PD-L1-expression was associated with an impaired survival when compared to patients with low PD-L1-expression when analyzed in a cohort of patients with pancreatic cancer who underwent surgical resection [30]. Just recently, Park et al. analyzed a cohort of 60 PDAC patients treated with FOLFIRINOX as first-line chemotherapy and demonstrated that patients with low levels of circulating PD-L1 at diagnosis showed better OS than those with high levels [17]. Moreover, in their analysis, circulating PD-L1 was found to be an independent prognostic factor for overall survival, highlighting that PD-L1 levels at diagnosis exhibit a prognostic value in pancreatic cancer [17]. In their study, Park et al. further demonstrated that circulating PD-L1 was higher at the time of disease progression than at the first response assessment, suggesting that the dynamics of PD-L1 levels are indicative of the disease course [17]. In our cohort, patients with low circulating PD-L1 levels demonstrated a strong trend towards an impaired survival compared to patients with higher levels. Similarly, patients with a further decrease in PD-L1 levels after tumor resection demonstrated an impaired prognosis compared to patients with an increase in PD-L1 levels. Although the differences failed to reach statistical significance in both analyses, our results argue for a potential role of circulating PD-L1 as a prognostic marker in patients with BTC, both when measured at the time-point of diagnosis and after surgery. Of note, concentrations of circulating PD-L1 were significantly negatively correlated with those of CCL1, CCL21, CCL25 and CCL26, which have been associated with (pro)malignant characteristics [21,22,23,24].

The data shown here relate exclusively to patients with resectable cholangiocarcinoma, i.e., patients in a relatively early stage of disease in which a cure can still be achieved. In recent years, beyond the classical therapeutic modalities such as resection or cytotoxic chemotherapy, a new therapeutic option using antibodies or small molecules has emerged for patients with nonresectable disease. However, it has become clear that these therapies are only effective in biomarker-selected patients. Therapy with IDH-1 inhibitors can only work in patients with corresponding mutations [31,32], and therapy with FGFR2 inhibitors is only effective in patients with a gene fusion or gene rearrangement [33,34]. These new data underline the high dynamics with which the clinical management of patients with cholangiocarcinoma is currently evolving [35,36]. The concept of biomarker analysis for therapy management and stratification of patients according to their prognosis will undoubtedly gain importance in the coming 5 years. Although the study shown here presents predominantly negative data, it joins a series of similar studies investigating the question of the useful application of biomarkers in BTC. Our study faces some important limitations. First, the study was conducted with a single-center design, and although strict and standardized criteria were applied for eligibility, surgical procedures and sample handling, this design warrants confirmation in a multicenter approach. Further, concentrations of sPD-L1 were very low and close to or even below the detection limit in some cases. The control population of healthy blood donors was not matched for age or gender. Moreover, our study included only patients in early disease stages undergoing surgical resection of BTC, and it remains unclear whether the data can be translated to patients in palliative disease stages. Finally, the number of included patients is relatively small; therefore, larger, multicenter clinical trials need to be conducted to provide further evidence on the pathophysiological and clinical role of circulating PD-L1 in BTC.

## 4. Patients and Methods

### 4.1. Study Design, Data Collection and Patient Characteristics

This observational cohort study was performed to analyze circulating levels of PD-L1 and their potential diagnostic and/or prognostic role in BTC patients undergoing surgical tumor resection. BTC patients who were admitted for tumor resection to the Department of Visceral and Transplantation Surgery at University Hospital RWTH Aachen were enrolled in this study between 2011 and 2015 (*n* = 73 patients, see Table 2 for detailed patient characteristics). Blood samples were collected prior to surgery and 6–7 days after BTC resection and centrifuged at 2000 g for 10 min, and serum samples were stored at −80 °C until use. Diagnosis of BTC was confirmed histologically in the resected tumor sample. As a control population, we analyzed a total of *n* = 42 healthy, cancer-free blood donors fulfilling the criteria for blood donation in Germany.

### 4.2. Determination of PD-L1 Serum Concentrations by ELISA

PD-L1 serum concentrations were analyzed using a commercial enzyme immunoassay (ELISA) according to the manufacturers’ instructions (No. SEA788Hu, Cloud-Clone Corp., Houston, TX, USA). The manufacturers specified the optimal detection range as 156 pg/mL–10 ng/mL, with the detection limit of 56 pg/mL.

### 4.3. Measurements of Cytokine Serum Levels

Serum levels of CCL1, CCL21, CCL25 and CCL26 were measured by multiplex immunoassay according to the manufacturer’s instruction using a Bio-Plex 200 system and Bio-Plex Manager 6.0 software (Bio-Plex Pro Human Chemokine Panel, #171AK99MR2, Bio-Rad, Hercules, CA, USA).

### 4.4. Statistical Analysis

Statistical analyses were performed as recently described [37,38]. Nonparametric data were compared using the Mann–Whitney U test and Kruskal–Wallis test. Related samples were compared with the Wilcoxon signed-rank test. The optimal diagnostic cut-off values for ROC curves were calculated using the Youden index (YI = sensitivity + specificity − 1). Kaplan–Meier curves display the impact on overall survival (OS). Log-rank test was performed to test for differences between groups. Optimal prognostic cut-off values were determined by fitting Cox proportional hazard models to the dichotomized survival status and the survival time and by defining the optimal cut-off as the point with the most significant split in the log-rank test. All statistical analyses were performed with SPSS 23 (SPSS, Chicago, IL, USA) and RStudio 1.2.5033 (RStudio Inc., Boston, MA, USA) [39]. A *p*-value of <0.05 was considered statistically significant (* *p* < 0.05; ** *p* < 0.01; *** *p* < 0.001).

## Figures and Tables

**Table 1:** Correlation analyses between preoperative PD-L1 and routine laboratory markers.

Parameter	PD-L1 Pre-OP	
	r_S_	*p*-Value
Sodium	−0.162	0.173
Potassium	0.146	0.222
Leukocytes	−0.073	0.543
Thrombocytes	−0.055	0.649
AST	0.068	0.568
ALT	0.086	0.550
Bilirubin	0.085	0.480
ALP	0.030	0.805
GGT	0.040	0.740
CRP	0.126	0.300
Creatinine	−0.092	0.440

AST, aspartate transaminase; ALT, alanine transaminase; ALP, alkaline phosphatase; CRP, C-reactive protein; GGT, γ-glutamyl transpeptidase.

**Table 2:** Characteristics of the study population.

Characteristic	
Healthy controls	42
BTC patients	73
Sex (%):	
male–female	52.8–47.2
Age (years, median and range)	68.0 (37–84)
BMI (kg/m^2^, median and range)	25.99 (19.15–46.36)
BTC characteristics (%):	
T1-T2-T3-T4	4.5–40.9–33.3–21.2
N0-N1	41.9–58.1
M0-M1	81.2–18.8
G2-G3	58.5–41.5
R0-R1	61.0–39.0
Tumor localization (%):	
intrahepatic CCA	34.2
perihilar CCA	37.0
distal	17.8
gallbladder carcinoma	11.0
Clinical performance status (%):	
ECOG 0-1-2	52.1-38.0-9.0
Laboratory parameters of BTC patients (median and range):	
WBC (cells/nL)	7.9 (2.9–21.6)
CRP (mg/L)	18.2 (0.0–230.0)
AST (U/L)	47.0 (18.0–1587.0)
ALT (U/L)	46.0 (10.0–1097.0)
GGT (U/L)	348 (36.0–2015.0)
ALP (U/L)	229.0 (53.0–1055.0)
Bilirubin (mg/dL)	1.0 (0.24–21.49)
Creatinine (mg/dL)	0.82 (0.43–1.9)
Haemoglobin (g/L)	12.5 (7.8–16.2)
Platelets (cells/nL)	275.0 (75.0–931.0)

## Data Availability

Datasets used in the current study are available from the corresponding author on reasonable request.

## Supplementary Materials

The following are available online at https://www.mdpi.com/article/10.3390/ijms22126569/s1, Figure S1: Post-operative serum PD-L1 does not correlate with clinicopathological characteristics, Figure S2. Serum PD-L1 negatively correlates with pro-malignant circulating cytokines. Table S1: Serum levels of various laboratory markers.
