# Supplementary material for: Levels of Circulating PD-L1 Are Decreased in Patients with Resectable Cholangiocarcinoma
Source: Int J Mol Sci. 2021 Jun 18;22(12):6569. doi: 10.3390/ijms22126569 (PMC8233871; doi:10.3390/ijms22126569)
Supplement: Supplementary file 1 [file ijms-22-06569-s001.zip › ijms-1242771-supplementary.pdf]

Supplementary Figure legends

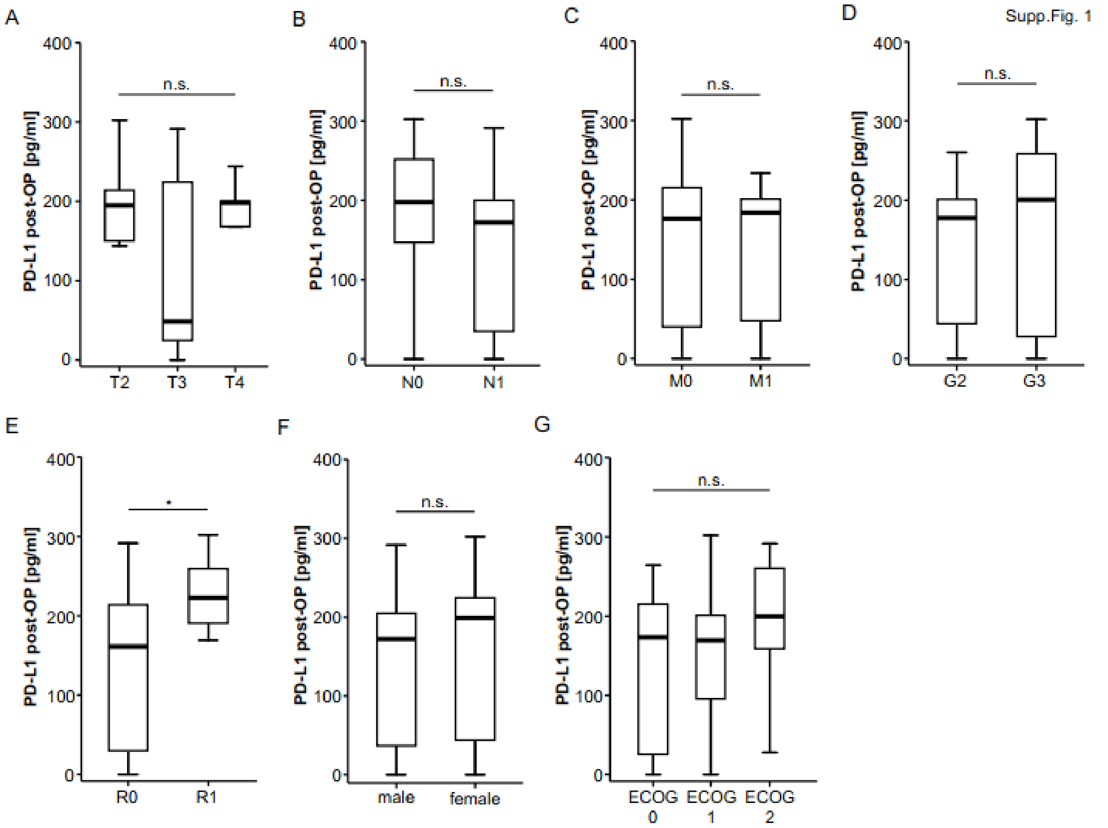

Supplementary Figure S1. Post-operative serum PD-L1 does not correlate with clinicopathological characteristics

Post-operative serum PD-L1 levels do not differ between different TNM status (A-C) and moderately or poorly differentiated tumors (D). Patients with a complete (R0) tumor resection have significantly lower post-operative PD-L1 serum levels. Post-operative PD-L1 serum levels are unaltered between male and female patients (F) and patients with different ECOG performance status (G).

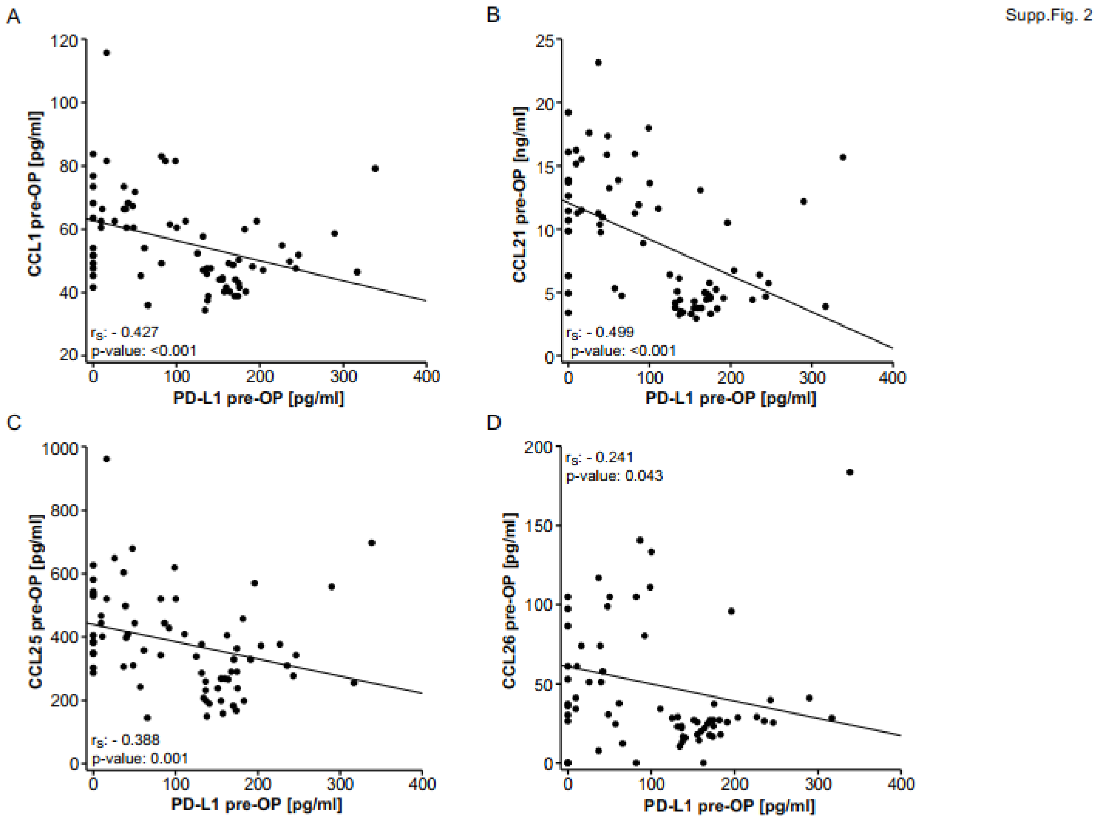

Supplementary Figure S2. Serum PD-L1 negatively correlates with pro-malignant circulating cytokines

Preoperative serum PD-L1 levels show a negative correlation with serum levels of CCL1 (A), CCL21 (B), CCL25 (C) and CCL26 (D).

Supplementary Tables

Supplementary Table S1: Serum levels of various laboratory markers

	**BTC patients** median [range]	**Healthy controls** median [range]
PD-L1 pre-OP [pg/ml]	110.08 [0.0-338.46]	182.3 [0.0-407.76]
PD-L1 post-OP [pg/ml]	177.49 [0.0-302.11]	-
CEA [µg/l]	3.25 [0.71-333.0]	1.30 [0.3-6.30]
CA19-9 [U/ml]	81.4 [0.60-22911.0]	5.35 [0.0-44.1]
Leucocyte count [cells/nl]	7.9 [2.90-21.60]	-
Platelets [cells/nl]	275.5 [75.0-931.0]	-
Sodium [mmol/l]	139.0 [131.0-146.0]	-
Potassium [mmol/l]	4.30 [2.90-5.50]	-
AST [U/l]	50.0 [18.0-1587.0]	30.0 [20.0-78.0]
ALT [U/l]	50.0 [10.0-1097.0]	24.0 [5.0-82.0]
GGT [U/l]	349.5 [36.0-2015.0]	19.0 [8.0-120.0]
ALP [U/l]	230.0 [53.0-1055.00]	69.0 [40.0-102.0]
CRP [mg/l]	18.5 [0.0-230.0]	
Bilirubin [mg/dl]	1.1 [0.24-21.49]	0.44 [0.10-1.46]
Creatinine [mg/dl]	0.83 [0.43-1.62]	-
CRP [mg/l]	18.5 [0.0-230.0]	
CCL1 [pg/ml]	51.68 [34.44-115.7]	
CCL21 [ng/ml]	6.73 [2.94-23.15]	
CCL25 [pg/ml]	358.04 [144.96-961.91]	
CCL26 [pg/ml]	28.76 [0.0-422.90]	

AST: aspartate transaminase, ALT: alanine transaminase, ALP: alkaline phosphatase, CA19-9: carbohydrate-Antigen 19-9, CEA: carcinoembryonic antigen, CCL: C-chemokine ligand, CRP: C-reactive protein, GGT: γ-Glutamyl transpeptidase, PD-L1: Programmed cell death 1 ligand 1
